# Exploring the Proteomic Signature of Diabetic Nephropathy: Implications for Early Diagnosis and Treatment

**DOI:** 10.3390/life15081312

**Published:** 2025-08-19

**Authors:** Duygu Sari-Ak, Fatih Con, Nazli Helvaci, Hayriye Ecem Yelkenci, Alev Kural, Ozgur Can, Mustafa Caglar Beker

**Affiliations:** 1 Department of Medical Biology, Hamidiye International School of Medicine, University of Health Sciences, Istanbul 34668, Turkey; 2 Clinic of Medical Biochemistry, Bakırkoy Dr. Sadi Konuk Training and Research Hospital, University of Health Sciences, Istanbul 34668, Turkey; con.fatih@gmail.com (F.C.); alevkural@hotmail.com (A.K.); 3 Department of Medical Biochemistry, Hamidiye School of Medicine, University of Health Sciences, Istanbul 34668, Turkey; tokazitaki.55@gmail.com; 4 Regenerative and Restorative Medical Research Center (REMER), Research Institute for Health Sciences and Technologies (SABITA), Istanbul Medipol University, Istanbul 34810, Turkey; heyelkenci@medipol.edu.tr (H.E.Y.); m.caglarbeker@gmail.com (M.C.B.); 5 Department of Nephrology, İstanbul Haydarpaşa Numune Training and Research Hospital, University of Health Sciences, Istanbul 34668, Turkey; canozgur62@hotmail.com; 6 Department of Physiology, School of Medicine, Istanbul Medeniyet University, Istanbul 34700, Turkey

**Keywords:** diabetic nephropathy, proteomics, biomarkers, mass spectrometry, inflammation, oxidative stress

## Abstract

Diabetic nephropathy (DN) is a leading cause of end-stage renal disease, characterized by progressive kidney dysfunction. Early detection and targeted therapies remain key challenges in managing DN. This study aims to identify proteomic alterations in DN patients compared to healthy controls, focusing on proteins involved in inflammation, oxidative stress, immune response, and metabolic dysregulation. Using mass spectrometry and advanced bioinformatics, we identified significant upregulation of proteins associated with platelet activation, immune regulation, and extracellular matrix remodeling, as well as downregulation of proteins linked to lipid metabolism, immune regulation, and structural stability. These findings highlight the molecular complexity of DN and suggest that altered protein expression plays a critical role in the progression of kidney damage. The identified proteins may serve as potential biomarkers for early diagnosis and therapeutic targets for DN. Our results underline the importance of proteomic analyses in advancing the understanding of DN pathogenesis and in developing strategies for personalized treatment to improve patient outcomes. Future research should focus on further elucidating these molecular mechanisms and their implications for clinical management.

## 1. Introduction

Diabetic nephropathy (DN) is a severe and common complication of diabetes mellitus (DM), significantly contributing to increased morbidity and mortality among affected individuals [1]. The global prevalence of diabetes, particularly in developing countries, is rising rapidly, and in the absence of effective preventive strategies, the incidence of DN is projected to increase accordingly [2]. DN develops in approximately 30% of patients with type 1 diabetes (DM1) and 40% of patients with type 2 diabetes (DM2), highlighting its clinical relevance across both types of diabetes [3,4]. Moreover, DN is the leading global cause of end-stage renal disease (ESRD), and the condition places a substantial burden on healthcare systems worldwide due to its prevalence and associated complications, underscoring the need for effective strategies to mitigate its impact [5].

The clinical progression of DN is characterized by persistent albuminuria (or an albuminuria excretion rate of >300 mg/d or 200 μg/min) measured at least twice within a three- to six-month interval. This condition is typically accompanied by a progressive decline in glomerular filtration rate (GFR) [6], often accompanied by elevated blood pressure, and ultimately leads to ESRD [7]. Despite advancements in diabetes management, DN remains a leading cause of renal failure, often requiring lifelong dialysis or kidney transplantation. However, early diagnosis and intervention can significantly slow disease progression and improve patient outcomes.

The pathogenesis of DN is highly complex and not fully understood, which limits the development of effective therapeutic strategies. Among the key contributors to DN progression are oxidative stress and inflammation, both of which play pivotal roles in mediating renal damage. The disease involves intricate interactions among metabolic, hemodynamic, and inflammatory pathways [8,9]. Hyperglycemia plays a central role by inducing oxidative stress and the overproduction of advanced glycation end products (AGEs), which disrupt cellular homeostasis and promote renal injury. The renin–angiotensin–aldosterone system (RAAS), particularly angiotensin II (Ang-II), contributes significantly to glomerular hypertension, inflammation, and fibrosis [10].

RAAS modulates intraglomerular hemodynamics and contributes to structural alterations within renal compartments, including podocyte function. Ang-II is increasingly recognized not only as a hemodynamic regulator but also as a pro-inflammatory and profibrotic cytokine that promotes kidney injury through both systemic and intrarenal RAAS activation, with oxidative stress playing a central and early role in the pathogenesis of DN by affecting nearly all renal cell types [10]. Hyperglycemia directly stimulates the synthesis of renin and Ang-II within mesangial cells, thereby amplifying intrarenal Ang-II activity under diabetic conditions [11,12]. Locally generated Ang-II induces multiple deleterious effects, including increased glomerular capillary pressure and permeability (leading to proteinuria), mesangial cell proliferation and hypertrophy, cytokine and extracellular matrix production, and macrophage infiltration, all of which contribute to renal inflammation and fibrosis [13].

Persistent inflammation is recognized as a central pathophysiological mechanism in DN, triggered by the underlying metabolic, hemodynamic, and biochemical imbalances characteristic of DM [14]. Furthermore, proinflammatory and fibrogenic cytokines secreted within the renal microenvironment can induce epithelial-to-mesenchymal transition, leading to excessive extracellular matrix accumulation and irreversible structural damage [15]. These findings highlight the role of inflammation not merely as a secondary effect but as a primary driver in the pathogenesis of DN.

Oxidative stress is a key contributor to the development of DN, arising from an imbalance between reactive oxygen species (ROS) and the body’s antioxidant defense mechanisms. Excessive ROS leads to damage in podocytes, mesangial cells, and endothelial cells, ultimately leading to proteinuria and interstitial fibrosis [16]. Inflammation further exacerbates renal injury, with proinflammatory cytokines such as interleukin-6 (IL-6) and tumor necrosis factor-alpha (TNF-α). These cytokines, along with other inflammatory mediators, promote macrophage infiltration and sustain a vicious cycle of inflammation and oxidative stress [8,17]. Hyperglycemia and AGEs activate transcription factors such as nuclear factor kappa B (NF-κB), which in turn upregulate proinflammatory cytokines and adhesion molecules, accelerating glomerular and tubular damage [18]. Additionally, protein kinase C (PKC) isoforms and their interactions with oxidative stress pathways contribute to mesangial expansion and glomerular hypertrophy [19].

The early detection and precise monitoring of DN are crucial for improving patient outcomes and reducing the burden of the disease [20]. Although microalbuminuria remains a widely used diagnostic marker, it has limited sensitivity and specificity, as kidney damage may begin before albumin becomes detectable in the urine [21,22]. As a result, there is growing interest in identifying novel biomarkers that provide earlier and more reliable indications of DN progression. These emerging biomarkers hold promise for transforming DN management by enabling earlier diagnosis, improved monitoring of disease progression, and timely therapeutic intervention [22]. Their integration into clinical practice may enhance diagnostic accuracy, support personalized treatment approaches, and slow disease progression. By detecting kidney damage at earlier stages, these tools have the potential to reduce the prevalence of advanced DN and associated complications, ultimately improving the prognosis for individuals with diabetes. Furthermore, these biomarkers contribute to a deeper understanding of DN pathogenesis, offering new avenues for targeted therapy and advancing precision medicine in nephrology [22,23].

Proteomics, the comprehensive study of proteins and their post-translational modifications, has become a vital tool for uncovering the mechanisms driving DN and identifying biomarkers for early detection, disease monitoring, and therapeutic intervention [24]. Unlike the fixed and static genome, the proteome is highly dynamic and reflects real-time cellular responses to various stimuli, including stress and disease. In the context of DN, proteomic analyses can reveal specific proteins and signaling pathways involved in glomerular damage, inflammation, and renal fibrosis. Advances in mass spectrometry (MS) and high-throughput technologies have greatly improved the ability to detect and quantify these proteins, facilitating the discovery of novel biomarkers and potential therapeutic targets.

This study aims to investigate proteomic differences between DN patients and healthy controls to identify proteins that may play a key role in the pathogenesis or progression of DN. The findings from this research could contribute to the development of new strategies for the diagnosis, treatment, and management of DN, ultimately improving patient outcomes.

## 2. Materials and Methods

### 2.1. Study Design and Participant Selection

This study was designed to investigate the proteomic alterations in DN patients by comparing blood samples from these patients to those from healthy controls. A total of 32 participants were included in the study, comprising 16 DN patients and 16 healthy control subjects.

#### 2.1.1. Inclusion Criteria

DN patients were selected based on established clinical diagnostic criteria, including persistent albuminuria (≥300 mg/day or ≥300 mg/g creatinine) confirmed in at least two out of three consecutive tests over a 3- to 6-month period, a reduced estimated glomerular filtration rate (eGFR < 60 mL/min/1.73 m^2^), and the exclusion of other primary or secondary kidney diseases. All patients had a documented history of DM. The DN group consisted of 8 women and 8 men, aged between 18 and 65 years.

Healthy controls were selected based on the absence of any known history of DM, DN, or other chronic kidney or systemic diseases. To objectively confirm their health status and rule out subclinical nephropathy or early-stage diabetes, all control individuals underwent laboratory evaluations, including normal fasting plasma glucose (<100 mg/dL), eGFR > 90 mL/min/1.73 m^2^, and the absence of albuminuria (urinary albumin < 30 mg/day or <30 mg/g creatinine). The healthy control group consisted of 9 women and 7 men, aged between 18 and 65 years.

#### 2.1.2. Ethical Approval

The study protocol was approved by the Hamidiye Scientific Research Ethics Committee of the University of Health Sciences, Turkey, under registration number 23/58. The study was conducted in accordance with the Helsinki Declaration and Good Clinical Practice guidelines. All participants provided written informed consent before enrollment in the study.

### 2.2. Sample Collection and Preparation

#### 2.2.1. Blood Sample Collection

Blood samples were obtained from both DN patients and healthy controls at a single time point. Around 5 mL of blood was drawn from each participant through venipuncture and collected in BD Vacutainer tubes containing K2 ethylenediaminetetraacetic acid (EDTA). Each tube contains approximately 9 mg of EDTA, which is adequate to prevent clotting and maintain the stability of the samples for later analysis.

#### 2.2.2. Sample Processing

Blood samples were immediately centrifuged at 2000× *g* for 15 min at 4 °C to separate plasma. The plasma samples were aliquoted and stored at −80 °C until they were used for a proteomic analysis.

### 2.3. Proteomic Analysis

#### 2.3.1. Protein Extraction and Quantification

To remove high-abundance proteins, including albumin, IgA, IgD, IgE, IgG (including light chains), IgM, alpha-1-acid glycoprotein, alpha-1-antitrypsin, alpha-2-macroglobulin, Apolipoprotein A1, fibrinogen, Haptoglobin, and Transferrin, which were present in high levels in the serum samples, the High-Select™ Top14 Abundant Protein Depletion Resin kit (A36370, Thermo Fisher Scientific, Waltham, MA, USA) was used. The removal of these proteins was performed using a depletion spin column following the manufacturer’s protocol. The sample was mixed with the resin slurry and incubated with gentle end-over-end agitation for 10 min at room temperature to ensure the binding of target proteins. After centrifugation at 1000× *g* for 2 min, the filtrate containing the depleted proteins in 10 mM phosphate-buffered saline (PBS) and 0.02% sodium azide (pH 7.4) was collected for further analysis. Protein concentration was determined using the Qubit Protein Assay Kit (Q33212, Invitrogen, Life Technologies, Carlsbad, CA, USA) with a Qubit 3.0 fluorometer (Q33216, Invitrogen, Life Technologies, Carlsbad, CA, USA). The fluorometer was calibrated with the standards provided in the assay kit, according to the manufacturer’s instructions. This fluorescence-based method ensures precise and reproducible protein concentration quantification, offering high sensitivity to protein content in the samples.

#### 2.3.2. Protein Digestion and Labeling

The samples were homogenized in 50 mM ammonium bicarbonate buffer (pH 7.8) (S2454-200ML, Sigma Aldrich, St. Louis, MO, USA) containing a protease inhibitor cocktail (ab270061, Expedeon, Heidelberg, Germany) and lysed at 95 °C using a protein extraction reagent kit (UPX Universal; Expedeon). After the lysis step, the samples were incubated for one hour at 4 °C. Protein concentrations were then determined using a Qubit 3.0 Fluorometer according to the manufacturer’s protocol. Following homogenization, the Filter-Aided Sample Preparation (FASP) Protein Digestion Kit (ab270519, Abcam, Cambridge, UK) was used to generate tryptic peptides as per the manufacturer’s instructions. A total of 50 μg in 30 μL protein samples were filtered through a 30 kDa cutoff spin column using 6 M urea. Subsequently, samples were alkylated with 10 mM iodoacetamide in the dark for 20 min at room temperature. The samples were then incubated overnight with MS grade trypsin protease (ratio 1:100, 90,057, Thermo Scientific, Waltham, MA, USA) at 37 °C. The next day, peptides were eluted from the columns and lyophilized. After lyophilization, the peptides were resuspended in 0.1% formic acid (1,002,642,510, Merck, Whitehouse Station, NJ, USA) and diluted to 100 ng/μL prior to injection into the Liquid Chromatography–Mass Spectrometry/Mass Spectrometry (LC-MS/MS) system, which consisted of an ACQUITY UPLC M-Class coupled with a SYNAPT G2-Si high-definition mass spectrometer (Waters, Milford, MA, USA).

#### 2.3.3. Mass Spectrometry Analysis

LC-MS/MS and protein identification were carried out with slight modifications to previously published protocols [25]. Briefly, the samples were loaded onto the ACQUITY UPLC M-Class, coupled with a SYNAPT G2-Si high-definition mass spectrometer (Waters). To equilibrate the columns, 97% of the mobile phase (0.1% formic acid in LC-MS grade water) was used, and the column temperature was maintained at 55 °C. Peptide separation was achieved using a 90 min gradient elution from the ACQUITY UPLC M-Class Symmetry C18 trap column (180 µm × 20 mm; 186007496, Waters) to the analytical column (ACQUITY UPLC M-Class HSS T3 Column, 100 Å, 1.8 µm, 75 µm × 250 mm; 186007474, Waters) at a flow rate of 0.400 μL/min, with a gradient from 4% to 40% acetonitrile (100029, Merck) containing 0.1% formic acid (*v*/*v*) [26]. MS and MS/MS scans in positive ion mode were performed sequentially with a 0.6-s cycle time. Collision energy (CE) was set at 10 V for low CE and 30 V for high CE. Ion mobility separation (IMS) was employed for ion separation, with wave velocity ramped from 1000 m/s to 55 m/s throughout the IMS cycle. The mobility trapping release time was set to 500 μs, with a trap height of 15 V. The IMS wave delay for mobility separation after trap release was 1000 μs. All ions within the 50–1900 m/z range were fragmented in resolution mode without any precursor ion preselection. A lock mass reference of 100 fmol/μL Glu-1-fibrinopeptide B (186007091-2, Waters, Milford, MA, USA) was used at a 60 s interval to monitor mass stability.

### 2.4. Bioinformatics and Statistical Analysis

Data analysis was performed using Progenesis-QI for Proteomics software (Version 1.0.0.2, Waters, https://www.nonlinear.com/progenesis/qi-for-proteomics/, accessed on 2 March 2025) to identify and quantify peptides. All identified proteins were assessed with at least two unique peptide sequences, and expression ratios were calculated. Pathway analysis was conducted using the Kyoto Encyclopedia of Genes and Genomes (KEGG), Reactome, and Gene Ontology (GO) enrichment through the Database for Annotation, Visualization, and Integrated Discovery (DAVID; https://david.ncifcrf.gov, v2024q4). Following pathway analysis, enrichment bubble plots were generated for the top pathways, displaying enrichment scores, *p*-values, and counts, using the SRplot online program (available at https://www.bioinformatics.com.cn/) [27]. Protein-protein interactions (PPI) were assessed using STRING (Search Tool for the Retrieval of Interacting Genes/Proteins) software (https://cn.string-db.org, USA), with a confidence level set at 0.7.

LC-MS/MS data were analyzed using IBM SPSS (Statistical Package for the Social Sciences, version 27, SPSS Inc., Chicago, IL, USA) and Python 3.14 (Python Software Foundation, 2023). Independent samples t-tests were performed, with a *p*-value of <0.05 considered statistically significant. To account for multiple comparisons, the Benjamini-Hochberg correction was applied, ensuring a more robust and reliable identification of significant results by controlling the false discovery rate. A log2 fold change threshold of <−1 and >1 was used to define significant differences between the groups.

## 3. Results

### 3.1. Study Population

In this study, we analyzed hematological and biochemical parameters in a cohort of 16 DN patients (eight women, eight men, aged 18–65 years) under nephrology follow-up, comparing them with healthy controls. The assessed hematological parameters included white blood cell (WBC) count, red blood cell (RBC) count, hemoglobin (HGB), hematocrit (HCT), platelets (PLT), mean corpuscular volume (MCV), mean corpuscular hemoglobin (MCH), mean corpuscular hemoglobin concentration (MCHC), and red cell distribution width (RDW) (Table 1).

The comprehensive biochemical analysis included measurements of serum creatinine, GFR, and hemoglobin A1c (HbA1c) in both patient and control groups, whereas it only included the spot urine albumin-to-creatinine ratio (ACR) and spot urine protein-to-creatinine ratio (PCR) for the patients (Table 1).

All patient samples were collected under clinically stable conditions, ensuring that the analyses reflected a consistent disease status. Matching the control group to the patient cohort in terms of age and sex further enhanced the reliability of the comparative analysis.

### 3.2. Comparative Proteomic Analysis Between Diabetic Nephropathy Patients and Healthy Controls

**Figure 1:**
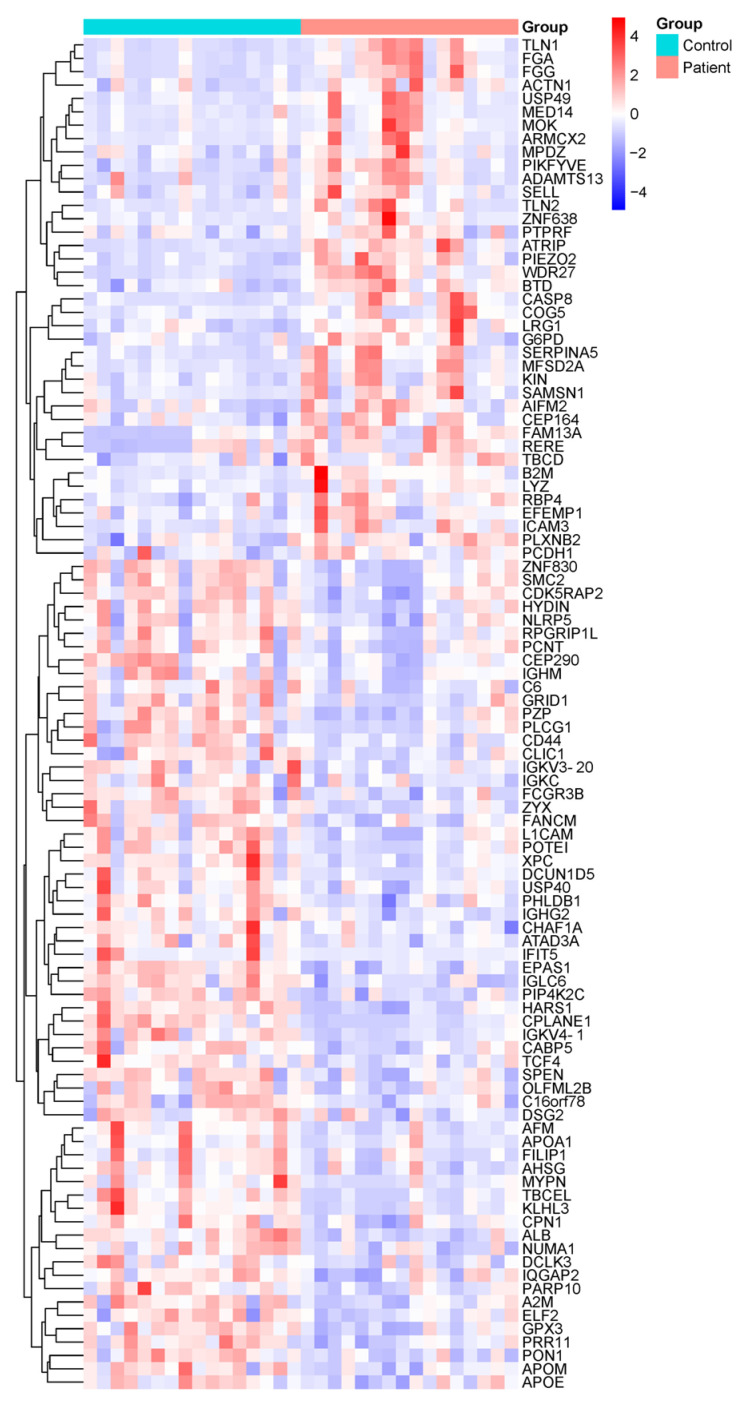
Overview of proteomic alterations in DN patients compared to healthy controls. Heatmap showing the hierarchical clustering of differentially expressed proteins in plasma samples from healthy control individuals (n = 16) and DN patients (n = 16), based on LC-MS/MS quantitative proteomic analysis. Color intensity represents the relative protein expression levels, with red indicating upregulation and blue indicating downregulation. Only proteins with statistically significant differences (*p* < 0.05) between groups are shown. Each row represents a single protein, and each column represents an individual sample. Protein abbreviations are listed in Supplementary Table S1 and correspond to the Human Protein Atlas (https://www.proteinatlas.org).

Following the depletion of highly abundant plasma proteins, comprehensive proteomic profiling was performed using LC-MS/MS on samples from both DN patients and healthy control subjects. A total of 500 distinct proteins were identified across all samples. Among these, 96 proteins demonstrated statistically significant differences in expression levels between the DN and control groups (*p* < 0.05) (Figure 1). Specifically, 37 proteins were significantly upregulated, while 59 were significantly downregulated in the DN group.

**Figure 2:**
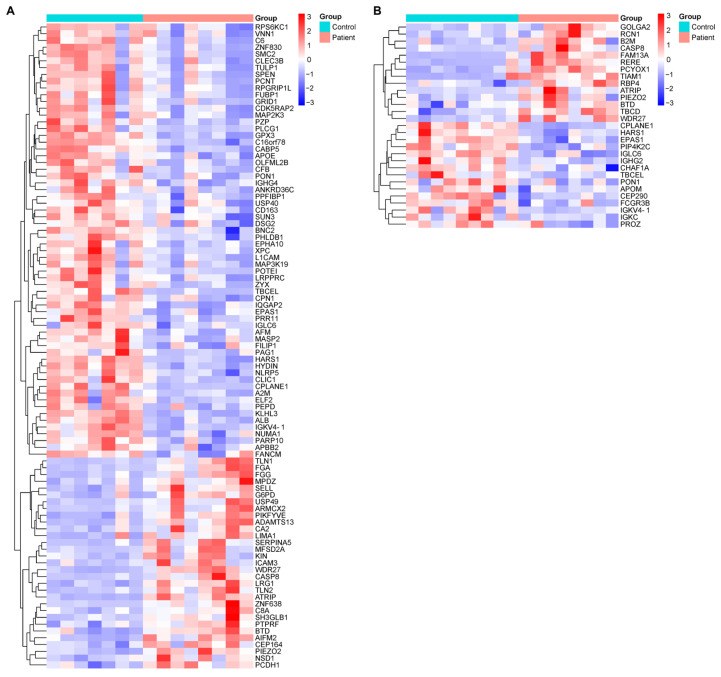
Heatmaps illustrating sex-stratified hierarchical clustering of differentially expressed proteins in plasma samples from DN patients and healthy controls. Protein quantification was performed using LC-MS/MS-based proteomic analysis. (**A**) Comparative expression profiles between male DN patients and healthy male controls. (**B**) Comparative expression profiles between female DN patients and healthy female controls. The color intensity represents the relative expression levels of proteins across the groups, with red indicating upregulation and blue indicating downregulation. Only proteins with statistically significant differences between patients and controls (*p* < 0.05) were included in each heatmap. Each row represents a single protein, and each column represents an individual sample. These sex-specific proteomic patterns may reflect differential pathophysiological responses or regulatory mechanisms in DN progression. Protein abbreviations are listed in Supplementary Table S1 and correspond with entries from the Human Protein Atlas (https://www.proteinatlas.org).

The comparative proteomic profiles of DN patients and healthy controls were analyzed separately for males and females to account for potential sex-specific differences in protein expression. These analyses revealed distinct proteomic alterations between male DN patients and healthy male controls, as well as between female DN patients and their healthy counterparts (Figure 2). The identified differentially expressed proteins (DEPs) may play critical roles in DN pathogenesis, progression, or compensatory mechanisms. By exploring sex-based variations, this study provides deeper insights into the molecular underpinnings of DN and may contribute to the identification of targeted biomarkers or therapeutic strategies tailored to male and female patients.

The proteins highlighted in Figure 1 and Figure 2, which exhibit significant changes in DN patients compared to the control group, are linked to specific biological pathways. These pathways and their associated proteins are outlined in Supplementary Table S2, which includes the results of the enrichment analysis. This table provides further insight into the biological relevance of the identified proteins and their roles in various molecular pathways, offering a deeper understanding of the molecular mechanisms involved in DN.

**Figure 3:**
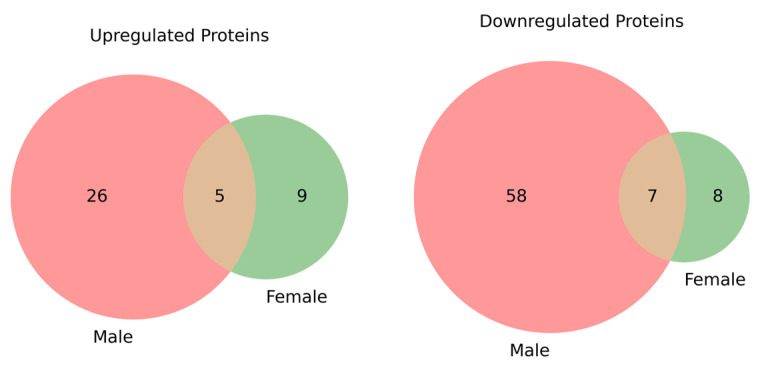
Venn diagrams showing the overlap of significantly (*p* < 0.05) upregulated and downregulated proteins between male and female groups. On the left, the upregulated proteins are shown, with 5 proteins common to both groups, 26 proteins unique to the male group, and 9 unique to the female group. On the right, the downregulated proteins are displayed, with 7 proteins common to both groups, 58 proteins unique to the male group, and 8 unique to the female group. These diagrams highlight the changes in protein expression between the DN patients and healthy controls, stratified by gender, and provide insight into the shared and distinct molecular alterations in male and female DN patients.

In our analysis, 96 proteins were significantly altered in male DN patients compared to male healthy controls, while 29 proteins showed significant changes in female DN patients compared to female healthy controls. Of these, 12 proteins were common to both comparisons, with 84 and 17 proteins uniquely altered in the male and female groups, respectively, as shown in Figure 3.

The expression of Golgin A 2 (GOLGA2), which was significantly upregulated in women, was found to be decreased in male patients compared to healthy males. Conversely, among men, the levels of LIM domain and actin-binding protein 1 (LIMA1), Selectin-L (SELL), Apoptosis-inducing factor mitochondria-associated 2 (AIFM2), and SH3 domain-containing GRB2-like endophilin B1 (SH3GLB1), all significantly upregulated, were reduced in female patients compared to healthy women. Additionally, the levels of Immunoglobulin heavy constant gamma 4 (IGHG4), Basonuclin-2 (BNC2), IQ motif-containing GTPase-activating protein 2 (IQGAP2), C-type lectin domain family 3 member B (CLEC3B), Sad1 and UNC84 domain containing 3 (SUN3), Ephrin type-A receptor 10 (EPHA10), and Far upstream element-binding protein 1 (FUBP1), which were significantly downregulated among men, were found to be elevated in female patients compared to healthy women. These proteins exhibited significant changes when comparing male and female patients.

Twelve common proteins were found to exhibit statistically significant changes inboth male and female groups when compared to their respective counterparts. Of these, five proteins (ATR-interacting protein (ATRIP), Caspase-8 (CASP8), Piezo-type mechanosensitive ion channel component 2 (PIEZO2), WD repeat domain 27 (WDR27), and Biotinidase (BTD)) were increased, while seven proteins (endothelial PAS domain protein 1 (EPAS1), immunoglobulin kappa variable 4-1 (IGKV4-1), ciliogenesis and planar polarity effector complex subunit 1 (CPLANE1), serum paraoxonase/arylesterase 1 (PON1), histidyl-tRNA synthetase 1 (HARS1), immunoglobulin lambda constant 6 (IGLC6), and tubulin folding cofactor E like (TBCEL)) were decreased. Among these proteins, WDR27, IGKV4-1, CPLANE1, HARS1, and TBCEL showed statistically significant changes when comparing male and female patients. These findings highlight shared molecular changes that may play a central role in the pathophysiology of DN, regardless of gender.

**Figure 4:**
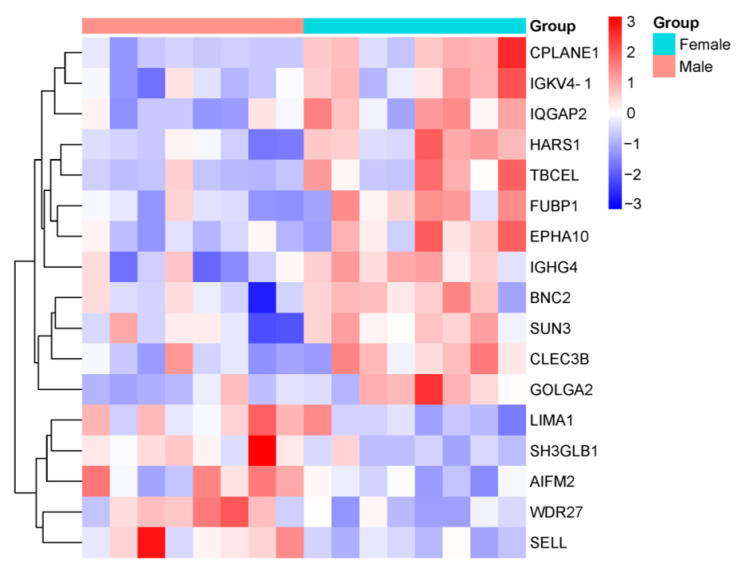
Heatmap showing the comparative proteomic profiles of DN patients, stratified by gender. The color intensity represents the relative expression levels of proteins across the groups, with red indicating upregulation and blue indicating downregulation. The heatmap highlights proteins that exhibit statistically significant differences (*p* < 0.05) between male and female patients.

The comparative proteomic profiles of male and female DN patients for these proteins are presented in Figure 4.

**Figure 5:**
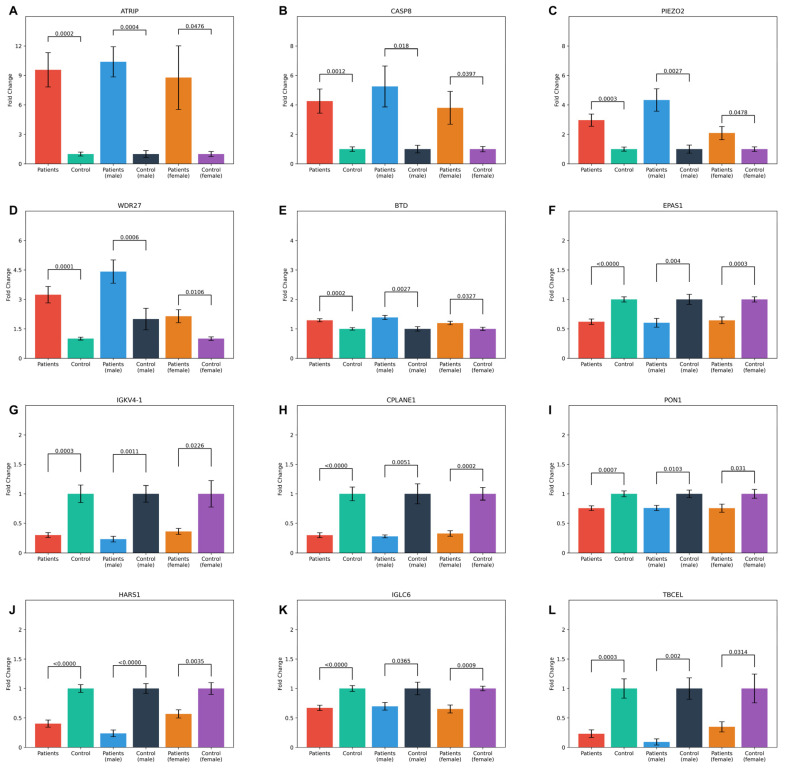
Bar plots showing the mean fold change values and standard error of the 12 common proteins in both patient and control groups across general, male, and female categories. The statistical significance of comparisons within each group is indicated by *p*-values. The proteins analyzed include (**A**) ATRIP, (**B**) CASP8, (**C**) PIEZO2, (**D**) WDR27, (**E**) BTD, (**F**) EPAS1, (**G**) IGKV4-1, (**H**) CPLANE1, (**I**) PON1, (**J**) HARS1, (**K**) IGLC6, and (**L**) TBCEL.

**Figure 6:**
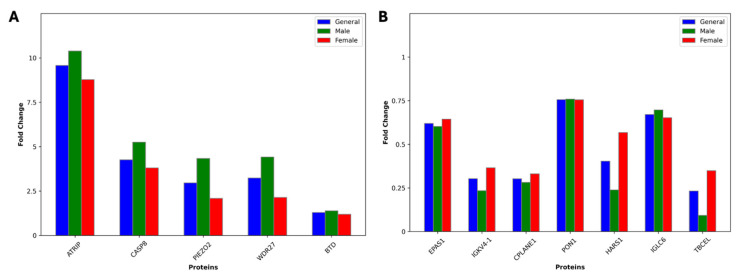
Bar plots showing the fold change values for 12 common proteins across the general, male, and female groups for (**A**) upregulated and (**B**) downregulated proteins. Fold change values were calculated between patient and control groups.

The comparison of protein expression profiles and fold change values between male and female patients and their respective control groups, which highlights 12 proteins with statistically significant differences, is presented in Figure 5 and Figure 6.

### 3.3. Differential Expression Analysis: Volcano Plots and Five-Fold Changes

**Figure 7:**
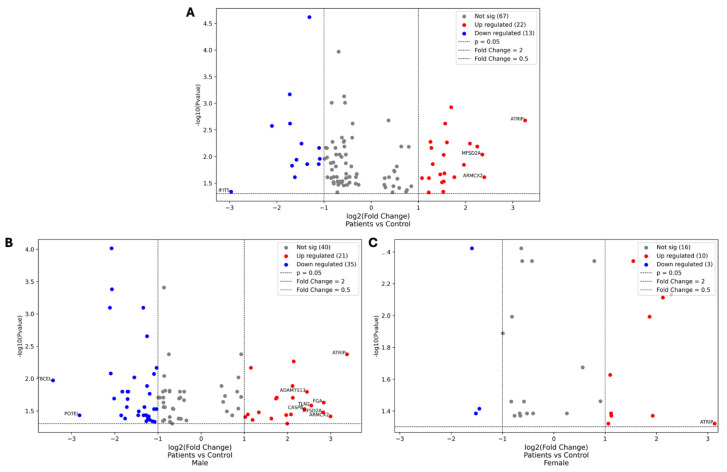
Volcano plots comparing the protein expression levels between DN patients and healthy controls. The first plot represents the overall comparison between DN patients and healthy controls (**A**), while the second and third plots illustrate gender-specific comparisons: male patients vs. male controls and female patients vs. female controls, respectively (**B**,**C**). Each dot represents a protein, with the x-axis showing the log2 fold change and the y-axis showing the −log10 *p*-value. Proteins that are significantly upregulated (red) and downregulated (blue) are highlighted, along with those that show at least a five-fold change in expression. These identified proteins are central to the discussion of the molecular mechanisms underlying DN and the pathogenesis of the disease.

The Volcano plots provided in Figure 7 illustrate the differential expression analysis of proteins between DN patients and healthy controls, with further comparisons stratified by gender: male patients vs. male controls and female patients vs. female controls.

These plots highlight the overall data distribution, emphasizing proteins that show significant changes in expression levels, including those with at least a five-fold increase or decrease.

In the comparison between DN patients and healthy controls, ATRIP was the most significantly upregulated protein, with a fold change of 9.58, while interferon-induced protein with tetratricopeptide repeats 5 (IFIT5) was the most significantly downregulated, with a fold change of 0.13. For male DN patients versus male controls, ATRIP showed the highest upregulation (fold change of 10.39), while TBCEL exhibited the most significant downregulation (fold change of 0.09). In female DN patients compared to female controls, ATRIP remained the most upregulated protein (fold change of 8.77), with CPLANE1 showing the greatest downregulation (fold change of 0.33).

### 3.4. Pathway Analysis and Protein–Protein Interaction Network in Diabetic Nephropathy Patients

**Figure 8:**
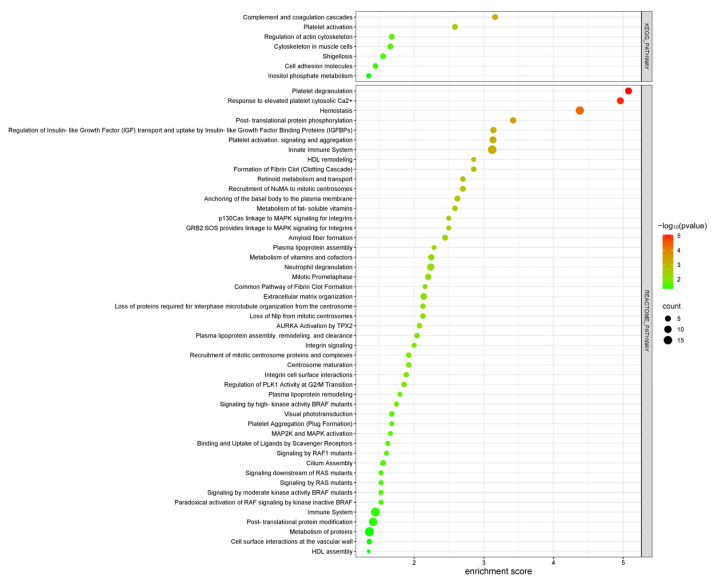
KEGG and REACTOME pathway enrichment analysis of differentially expressed proteins in DN patients compared to healthy controls. The bubble plot displays key signaling pathways, with the x-axis and the color gradient indicating statistical significance (−log10 *p*-value) and the count bar showing the number of proteins involved in each pathway.

Pathway analysis was conducted using the DAVID tool, focusing on KEGG and Reactome pathways to identify key signaling pathways involved in DN, which revealed several enriched pathways that play critical roles in DN pathology and provide insights into the molecular mechanisms underlying disease progression (Figure 8). Among the KEGG pathways, “Platelet activation” and “Complement and coagulation cascades” were prominently enriched, indicating their substantial roles in inflammatory and thrombotic processes within DN. These pathways emphasize the heightened pro-inflammatory and pro-thrombotic state associated with DN progression. Within the Reactome pathways, notable enrichment was observed in pathways such as “Platelet activation, signaling and aggregation”, “Hemostasis”, “Post-translational protein modification”, “Innate Immune System”, and “Metabolism of proteins”. These pathways underscore the significance of cellular communication, protein metabolism, and intracellular signaling in the context of DN pathophysiology. These pathways also reflect the significant involvement of thrombotic processes and blood clotting, pointing to a disrupted vascular environment in DN. Additionally, the regulation of protein function and cellular responses underscores the importance of intracellular signaling in DN progression. The activation of the immune system and the associated inflammation also play a critical role in the disease’s development.

Overall, these findings suggest that the disruption of immune responses, signaling pathways, and protein metabolism plays a central role in the molecular mechanisms driving DN. The enrichment of pathways involved in platelet activation, hemostasis, protein modification, and immune system regulation highlights the complex interplay of inflammatory, thrombotic, and metabolic processes in the progression of the disease.

**Figure 9:**
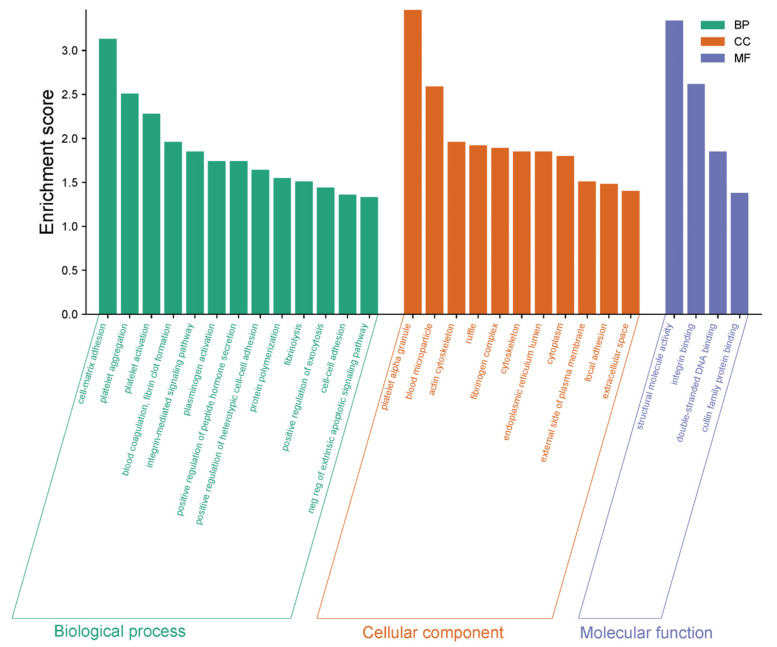
GO enrichment analysis of biological processes (BP), cellular components (CC), and molecular functions (MF) comparing DN patients and healthy controls. The x-axis represents enriched terms for each GO category, while the y-axis indicates the enrichment score. Green bars correspond to BP, orange bars to CC, and blue bars to MF categories. Key enriched terms highlight the significance of inflammatory responses, cytoskeletal reorganization, and molecular interactions in DN pathophysiology.

GO enrichment analysis comparing DN patients with healthy controls highlights significant alterations in biological processes (BP), cellular components (CC), and molecular functions (MF), providing a comprehensive view of the molecular mechanisms underlying DN (Figure 9). Among the biological processes, complement activation, platelet aggregation, and blood coagulation emerged as key pathways, underscoring the heightened inflammatory and vascular complications associated with DN. Additionally, processes like the positive regulation of peptidase activity and protein phosphorylation reflect disrupted enzymatic activity and signaling mechanisms that contribute to disease progression. From a cellular component perspective, platelet alpha granules, blood microparticles, and the actin cytoskeleton were prominently enriched, suggesting significant cytoskeletal reorganization and changes in cellular compartment interactions, which are crucial for maintaining cellular integrity and function in DN. At the molecular function level, structural molecule activity, integrin binding, and double-stranded DNA binding were significantly represented, pointing to the importance of PPI, structural support, and genomic stability in the pathophysiology of DN. These findings collectively provide crucial insights into the inflammatory responses, cellular interactions, and molecular disruptions that drive DN, offering potential pathways for therapeutic targeting and biomarker discovery.

**Figure 10:**
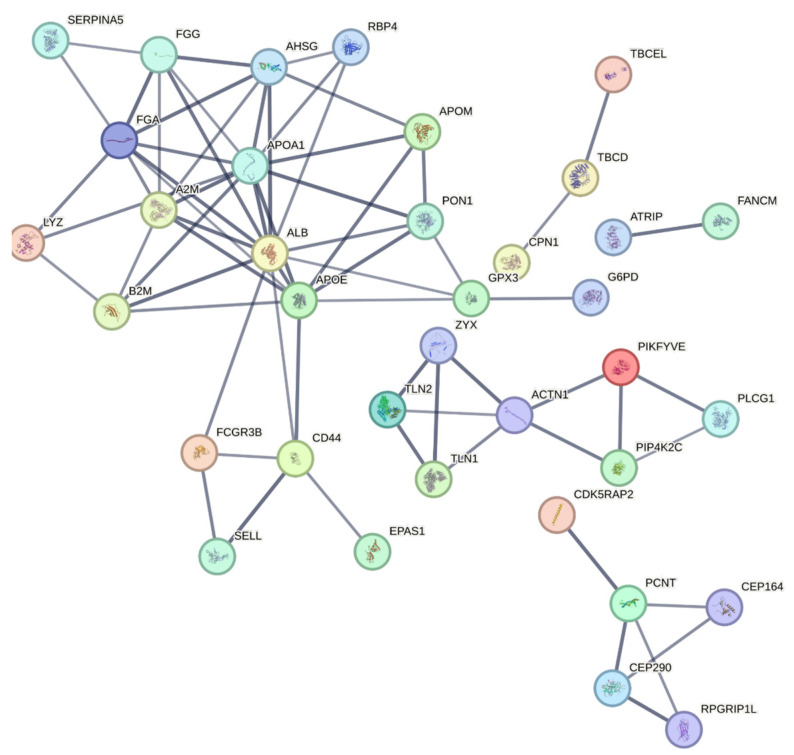
PPI network generated using STRING with a confidence score of 0.7 showing interactions between significantly altered proteins. Central proteins with extensive connections, such as ALB, APOA1, A2M, FGA, FGG, AHSG, APOE, TLN1, TLN2, and ZYX, are emphasized for their potential roles in DN’s molecular mechanisms and as therapeutic targets. This network highlights the complex interplay of signaling pathways and biological processes in diabetic nephropathy.

To further elucidate the molecular mechanisms underlying DN, a PPI network for the diabetic nephropathy group was constructed using the STRING database, with a high confidence score of 0.7. Among the key proteins identified, albumin (ALB), Apolipoprotein A1 (APOA1), alpha-2-macroglobulin (A2M), fibrinogen alpha chain (FGA), fibrinogen gamma chain (FGG), alpha-2-HS-glycoprotein (AHSG), Apolipoprotein E (APOE), Talin 1 (TLN1), Talin 2 (TLN2), and Zyxin (ZYX) emerged as central hubs (Figure 10). The analysis highlights the crucial role of several proteins in key processes related to DN. Cell adhesion is notably regulated by proteins like FGA, FGG, CD4, SELL, TLN1, and TLN2, which are involved in cellular interactions that are essential for maintaining tissue integrity and vascular function. These proteins play a significant role in adhesion processes, potentially contributing to the vascular abnormalities observed in DN. In lipid metabolism, proteins such as APOA1, APOE, and Apolipoprotein M (APOM) are vital for high-density lipoprotein (HDL) particle clearance and plasma lipoprotein particle assembly. These proteins are central to the regulation of lipid homeostasis, with potential implications for the dyslipidemia commonly seen in DN. The complement and coagulation cascades involve proteins like ALB, A2M, FGA, FGG, AHSG, and Serpin family A member 5 (SERPINA5). These proteins are integral to the inflammatory and thrombotic processes that are elevated in DN. Their roles in the immune response and blood coagulation emphasize the heightened pro-inflammatory and pro-thrombotic state that accelerates DN progression. Additionally, proteins such as FGA, FGG, A2M, ALB, and AHSG are involved in hemostasis and the dissolution of fibrin clots, further indicating their importance in blood clot regulation and the inflammatory response in DN. This suggests that disturbances in these proteins may contribute to the vascular dysfunction and clotting abnormalities seen in DN.

These proteins not only represent key drivers of DN progression but also offer potential therapeutic targets, as modulating their activity could impact multiple pathways simultaneously, providing strategic opportunities for intervention. Together, these proteins highlight the multifaceted nature of DN, which involves cell adhesion, lipid metabolism, blood coagulation, and inflammatory responses. Their roles in these processes suggest potential therapeutic targets for modulating these pathways and improving clinical outcomes for DN patients.

## 4. Discussion

This study provides a comprehensive proteomic characterization of molecular alterations associated with DN, utilizing high-resolution LC-MS/MS-based analysis. Through the identification and quantification of 500 plasma proteins, we uncovered 96 DEPs between DN patients and healthy controls. These DEPs are involved in diverse biological processes, many of which are directly implicated in the pathogenesis and progression of DN, including inflammation, oxidative stress, metabolic dysregulation, vascular remodeling, and cytoskeletal dynamics.

Among the downregulated proteins, enrichment analysis revealed significant associations with immune regulation, lipid metabolism, and cytoskeletal integrity. Specifically, components of the complement and coagulation cascades, HDL remodeling, and microtubule polymerization pathways were suppressed in DN patients. These findings suggest impaired innate immune surveillance, altered lipid transport, and compromised cellular organization. A reduction in complement activity may render renal tissues more susceptible to infection and inflammatory injury, while HDL dysfunction could promote lipid accumulation and oxidative stress. Similarly, disturbances in microtubule dynamics may hinder cell division, intracellular transport, and overall cytoskeletal integrity—factors that can accelerate glomerular damage and interstitial fibrosis.

Conversely, upregulated proteins were predominantly involved in pro-inflammatory, pro-thrombotic, and stress response pathways, including platelet activation, Toll-like receptor signaling, and cell adhesion. This shift toward immune activation and enhanced vascular reactivity reflects an environment of chronic inflammation and endothelial dysfunction, which are hallmark features of DN. The upregulation of proteins involved in cytoskeletal remodeling, exosome production, and intercellular communication further supports the presence of dynamic tissue remodeling in response to sustained diabetic injury.

Interestingly, the simultaneous downregulation of proteins linked to antibody production and adaptive immunity points to an imbalance between innate and adaptive immune responses. This may contribute to a dysfunctional immune microenvironment characterized by chronic low-grade inflammation coupled with impaired resolution mechanisms. Additionally, changes in exosome-associated proteins and lipid transporters suggest an ongoing effort by renal cells to adapt to metabolic stress through altered vesicle trafficking and nutrient handling. The upregulation of pathways linked to viral infections and cellular stress responses indicates a heightened activation of antiviral mechanisms and cellular adaptations, likely as reactions to inflammation and immune dysregulation in DN. However, these adaptations might also play a role in the pathological aspects of DN, underscoring the importance of investigating immune regulation, lipid metabolism, and cellular stability as potential therapeutic targets for managing DN.

A closer inspection of the most significantly altered proteins revealed several candidates with strong mechanistic links to DN. For instance, ATRIP was notably upregulated, potentially indicating increased DNA repair activity in response to oxidative stress-induced damage in podocytes—cells known to be particularly vulnerable in DN. Family with sequence similarity 13 member A (FAM13A), which is involved in adipocyte differentiation and lipid metabolism, may reflect maladaptive metabolic responses that are closely linked to insulin resistance and renal lipotoxicity.

In the analyses between the DN and control groups, the proteins EPAS1, IGKV4-1, PON1, and HARS1 were significantly decreased, while ATRIP, FAM13A, CASP8, PIEZO2, BTD, and ADAM metallopeptidase with thrombospondin type 1 motif 13 (ADAMTS13) were significantly increased. These proteins are thought to play important roles in DN pathophysiology.

EPAS1, also known as Hypoxia-Inducible Factor 2 Alpha (HIF-2α), is a transcription factor that orchestrates cellular responses to hypoxic conditions by regulating genes involved in angiogenesis, erythropoiesis, and vascular remodeling. In the context of DN, its downregulation may signify impaired hypoxia-driven signaling pathways, potentially disrupting the kidney’s ability to adapt to reduced oxygen availability. This dysfunction could enhance oxidative stress, promote endothelial injury, and accelerate fibrotic remodeling in renal tissues. Supporting this, Luque et al. demonstrated that endothelial-specific deficiency of EPAS1 in a hypertensive mouse model led to the loss of glomerular endothelial cell fenestration, increased endothelial swelling, and progressive glomerulosclerosis, including the development of focal segmental glomerulosclerosis (FSGS) [28]. These alterations exacerbated proteinuria and compromised the glomerular filtration barrier, despite comparable blood pressure responses, highlighting EPAS1’s protective role in glomerular structure and function. Its downregulation in DN patients suggests a diminished capacity for vascular adaptation, which may contribute to progressive kidney injury.

IGKV4-1, a variable region of the immunoglobulin kappa light chain, plays a critical role in the adaptive immune response, particularly in antigen recognition and antibody production. Previous studies have reported increased IGKV4-1 expression during advanced stages of DN, implicating it in immune complex formation and glomerular inflammation [29]. However, our study identified a significant downregulation of IGKV4-1 in DN patients, potentially reflecting immune suppression or dysregulation at earlier stages of the disease. This reduction may impair effective humoral immunity, increasing susceptibility to infection and dampening immune surveillance. The stage-specific expression patterns of IGKV4-1 suggest a dynamic role in DN pathophysiology, where early suppression could precede later overactivation and contribute to immune-mediated tissue damage and fibrosis.

PON1, an enzyme primarily associated with HDL, plays a protective role in lipid metabolism by hydrolyzing lipid peroxides and preventing oxidative damage to lipoproteins and cell membranes. Its antioxidant function is particularly relevant in diabetes-related complications, where increased levels of AGEs promote oxidative stress. Ikeda et al. reported decreased PON1 activity in patients with non-insulin-dependent diabetes mellitus, particularly in those with microvascular complications [30], while Zhou et al. found an inverse correlation between PON1 activity and AGEs in diabetic patients with proteinuria [31]. In our study, the significant downregulation of PON1 in DN patients may indicate impaired antioxidant defense in the kidney, contributing to enhanced oxidative damage, endothelial dysfunction, and progression of glomerular injury. Reduced PON1 activity may also compromise HDL function, exacerbating dyslipidemia and vascular inflammation. Although promising as a biomarker for chronic kidney disease (CKD) progression, the diagnostic utility of PON1 requires further validation in larger, well-characterized patient cohorts [32].

HARS1 is a key enzyme in protein biosynthesis, responsible for charging tRNA with histidine during translation. It plays a central role in maintaining protein homeostasis and supporting cellular function. While HARS1 mutations have been implicated in peripheral neuropathy due to impaired protein synthesis and increased cellular stress [33], its downregulation in DN may suggest a similar mechanism within renal tissues. Reduced HARS1 expression could reflect compromised translational capacity, limiting the kidney’s ability to maintain cellular repair and regeneration under diabetic conditions. This may result in increased cellular vulnerability, impaired structural maintenance, and accelerated disease progression.

ATRIP, a critical mediator of the DNA damage response, was significantly upregulated. ATRIP facilitates the recognition and repair of DNA double-strand breaks, particularly under cellular stress. In DN, chronic hyperglycemia and oxidative stress contribute to the accumulation of DNA damage in renal podocytes, which are terminally differentiated cells with limited regenerative capacity. The observed upregulation of ATRIP in DN patients likely reflects an enhanced DNA repair response aimed at preserving genomic stability in damaged podocytes, thus representing a cellular adaptation to diabetic stress [34].

FAM13A is involved in the regulation of inflammation and metabolic homeostasis. It plays a role in adipocyte differentiation, fat distribution, and insulin sensitivity. FAM13A has previously been associated with cardiometabolic diseases and obesity-related conditions [35]. In this study, its upregulation may indicate a response to metabolic dysregulation in DN, particularly related to altered lipid metabolism and increased visceral fat accumulation—both of which are known contributors to renal dysfunction and cardiovascular risk in diabetic populations [36,37]. These findings support the hypothesis that FAM13A may be involved in maladaptive metabolic remodeling in DN.

CASP8, a key enzyme regulating programmed cell death pathways, including apoptosis, necroptosis, and pyroptosis, was also upregulated. CASP8 is known to mediate the activation of downstream effector caspases, promote cytokine production, and facilitate immune cell recruitment. Its increased expression in DN may reflect heightened inflammatory signaling, contributing to immune cell infiltration, renal inflammation, and fibrosis—all hallmark features of DN progression [38,39,40]. Furthermore, CASP8-mediated apoptosis in renal cells, such as podocytes and tubular epithelial cells, may exacerbate tissue damage and loss of renal function.

PIEZO2 is a mechanosensitive ion channel involved in sensing mechanical stimuli and regulating cellular responses to mechanical stress. In the diabetic kidney, increased intraglomerular pressure and extracellular matrix accumulation impose mechanical strain on mesangial and podocyte cells. Previous studies in diabetic models have shown that PIEZO2 expression is upregulated in mesangial cells and is associated with increased fibronectin production, contributing to glomerulosclerosis [41]. Our findings support the hypothesis that PIEZO2 plays a mechanotransductive role in DN and may drive fibrosis and structural remodeling in response to glomerular stress.

BTD, an enzyme essential for recycling biotin—a vital coenzyme in fatty acid synthesis, gluconeogenesis, and amino acid metabolism—was found to be upregulated. BTD ensures the availability of free biotin by cleaving biocytin and supports cellular metabolic flexibility under stress conditions. A previous proteomic study identified BTD as a promising urinary biomarker for diabetic kidney disease (DKD), demonstrating its discriminatory power between DKD and non-DKD patients [42]. In DN, elevated BTD expression may reflect an increased cellular demand for biotin to support adaptive metabolic responses under chronic hyperglycemia and oxidative stress. This suggests a compensatory mechanism aimed at maintaining energy balance and metabolic stability in diseased renal tissue.

ADAMTS13, a metalloprotease responsible for cleaving ultra-large von Willebrand factor (vWF) multimers, plays a crucial role in regulating platelet aggregation and preventing thrombotic microangiopathy. Domingueti et al. explained that the imbalance between ADAMTS13 and VWF contributes to vascular damage, promoting thrombosis and endothelial dysfunction, which are key factors in the progression of both nephropathy and atherosclerotic cardiovascular disease [43]. Their study suggested several potential mechanisms for the reduced ADAMTS13 levels in DN patients, including decreased synthesis due to the inflammatory environment, proteolytic degradation by increased plasma proteases such as thrombin and plasmin, and the loss of ADAMTS13 in the urine due to kidney dysfunction [43]. However, the upregulation observed in our study may represent a compensatory response aimed at counteracting excessive vWF levels and maintaining vascular homeostasis. Alternatively, it may reflect an adaptive mechanism to limit thrombotic and inflammatory damage within the glomerular microvasculature. Nevertheless, whether this increase is sufficient to overcome the prothrombotic state associated with DN remains unclear and warrants further investigation.

In our study, WDR27, major facilitator superfamily domain containing 2A (MFSD2A), and armadillo repeat containing X-linked 2 (ARMCX2) were significantly upregulated, suggesting their involvement in cellular adaptation to DN. WDR27 is associated with cell signaling and cytoskeletal organization; its increased expression may reflect a compensatory response to cellular stress and structural disruption. Similarly, the upregulation of MFSD2A, a key protein in lipid transport and blood-brain barrier maintenance, suggests altered lipid handling, potentially as a mechanism to address energy imbalances in DN. ARMCX2, which regulates mitochondrial dynamics, was also elevated, possibly indicating mitochondrial dysfunction and impaired intracellular energy regulation in the diabetic kidney. Collectively, these findings point to stress-related adaptive responses aimed at preserving cellular integrity under diabetic conditions. In contrast, IFIT5, TBCEL, and CPLANE1 were significantly downregulated. IFIT5, an interferon-induced protein involved in antiviral defense and immune regulation, showed reduced expression, suggesting a weakened innate immune response that could worsen inflammation and immune imbalance in DN. TBCEL, which regulates microtubule dynamics, was also downregulated, potentially compromising cytoskeletal organization and contributing to renal instability and tissue remodeling. Similarly, CPLANE1, a protein involved in ciliogenesis and maintaining cellular polarity, showed reduced expression, indicating disruptions in cilia-mediated signaling and epithelial structure. These downregulations highlight the interplay between immune and structural dysfunction in DN pathogenesis.

Proteomics plays a crucial role in uncovering the complex molecular mechanisms underlying DN. By identifying disease-specific biomarkers and altered molecular pathways, proteomic studies not only enhance our understanding of DN pathophysiology but also support the development of early diagnostic tools, personalized therapeutic strategies, and targeted interventions to improve clinical outcomes. Si et al. [44] demonstrated that integrating multi-omics data can reveal novel therapeutic targets in CKD, including DN. Their study highlighted proteins such as fibroblast growth factor 5 (FGF5), Complement C4-A (C4a), and Soluble Receptor for Advanced Glycation End Products (sRAGE) as potential biomarkers and therapeutic targets. Similarly, Zhang et al. [45] applied proteome-wide Mendelian randomization and colocalization analyses to identify plasma proteins—such as Cerebellin-1 (CBLN1), collagen alpha-2(VI) chain (COL6A2), inter-alpha-trypsin inhibitor heavy chain H3 (ITIH3), and transforming growth factor-beta-induced protein ig-h3 (TGFBI)—that exhibit causal relationships with disease progression, suggesting their relevance in drug development for DN. Van Roy and Speeckaert [46] emphasized the promise of targeted proteomics and metabolomics in improving early detection and personalized treatment for DKD. However, they noted that clinical application remains limited due to challenges such as the need for robust validation, technological complexity, and a lack of standardized workflows for clinical integration. Hill et al. [47] further underscored the global rise in diabetes and DKD, advocating for the inclusion of comorbidity, environmental, and demographic variables in multi-omics research to address health disparities related to ethnicity, sex, and socioeconomic status. Hu et al. [48] highlighted how combining genomics, transcriptomics, proteomics, and metabolomics has improved our understanding of immune and inflammation-related mechanisms in DKD. Their findings emphasized the contribution of immune cells—particularly macrophages and T lymphocytes—to disease progression and demonstrated the potential of multi-omics to guide treatment strategies tailored to the heterogeneous nature of DN.

Among current analytical platforms, LC-MS/MS is widely regarded as the gold standard in proteomics due to its superior sensitivity, specificity, and multiplexing capabilities. Compared to conventional immunoassays, LC-MS/MS enables more accurate detection of protein isoforms, post-translational modifications, and low-abundance proteins, making it particularly valuable for both early disease detection and therapeutic monitoring. Over the past decade, proteomics studies utilizing LC-MS/MS have led to the identification of thousands of candidate biomarkers across a wide spectrum of diseases, including cancer, metabolic disorders, and neurodegenerative conditions [49].

Despite these advances, several technical and logistical challenges continue to hinder the routine clinical implementation of LC-MS/MS-based proteomics [50,51]. A lack of standardized protocols for sample preparation and data analysis contributes to inter-laboratory variability and limited reproducibility. Moreover, the integration of MS-based workflows into existing clinical infrastructures is often constrained by incompatibility with laboratory information systems (LIS), which impedes automation and seamless result reporting. Some mass spectrometry platforms also suffer from limited throughput, while data interpretation requires specialized bioinformatics expertise. Additional barriers include a shortage of well-characterized biospecimens and validated reference standards necessary for assay development and external quality control. Furthermore, the limited availability of high-affinity reagents restricts the translation of proteomic discoveries into widely used clinical formats, such as enzyme-linked immunosorbent assay (ELISA). Nonetheless, promising developments are emerging. The incorporation of machine learning into proteomic data analysis is facilitating biomarker prioritization and personalized risk prediction. Importantly, the translation of high-confidence mass spectrometry findings into immunoassay-based platforms—such as ELISA or rapid diagnostic kits—could bridge the gap between research and clinical application, especially in point-of-care settings.

This study acknowledges a key limitation: the relatively small sample size, which may affect the generalizability of the results. Although the proteomic data obtained were biologically relevant and consistent with previous findings, the limited cohort may not fully reflect the heterogeneity of the broader DN population. As this was a discovery-phase study, the primary aim was to explore the proteomic landscape of DN and generate testable hypotheses for future validation. Small sample sizes are a common constraint in clinical proteomics, largely due to high analytical costs, complex workflows, and the need for high-quality, well-preserved samples. Despite these limitations, our findings offer valuable preliminary insights into the molecular alterations associated with DN. Future investigations involving larger and more diverse cohorts will be essential to validate these findings, confirm the proposed biomarkers and pathways, and support their potential application in clinical settings.

By comparing these findings with previous studies, we gain a more profound understanding of the molecular mechanisms driving DN and the potential role of these proteins as biomarkers for disease progression or as therapeutic targets. The notable fold changes observed in these proteins suggest that they could be crucial in the development and progression of DN.

## 5. Conclusions

This study provides a comprehensive analysis of the molecular alterations associated with DN, identifying key proteins and pathways involved in disease progression. The observed differences in proteomic profiles between DN patients and healthy controls highlight the ongoing pathological processes characteristic of DN, particularly those related to inflammation, oxidative stress, immune dysregulation, and metabolic dysfunction. Our results underscore the multifactorial nature of DN, implicating a complex network of interconnected biological pathways that contribute to progressive renal injury and functional decline.

The identification of differentially expressed proteins associated with DN progression offers valuable biomarker candidates for early diagnosis, disease monitoring, and therapeutic response assessment. These insights support the development of more precise and personalized treatment strategies that target the specific molecular disturbances underlying DN.

In conclusion, this study advances our understanding of the proteomic landscape of DN and emphasizes the need for integrative and pathway-oriented therapeutic approaches. Future research should focus on validating the identified biomarkers in larger and more diverse cohorts, as well as developing targeted interventions that address the core molecular mechanisms driving DN pathogenesis. Such efforts may ultimately improve clinical outcomes and enhance the quality of life for patients affected by this progressive renal complication.

## Figures and Tables

**Table 1:** Biochemical parameters measured in the control group, and DN patients. Data are presented as mean ± standard deviation (SD) for each group. Reference ranges are provided for comparison.

Parameter	Control Group (Mean ± SD)	Patient Group (Mean ± SD)	Reference Range
WBC (×10^9^/L)	6.45 ± 1.45	8.73 ± 2.13	4.0–11.0
RBC (×10^12^/L)	4.62 ± 0.34	4.45 ± 0.64	4.2–6.1
HGB (g/dL)	13.28 ± 1.63	12.05 ± 2.26	12.0–17.5
HCT (%)	40.18 ± 4.38	37.28 ± 6.54	36–53
PLT (×10^9^/L)	255.5 ± 67.92	278.5 ± 69.32	150–450
MCV (fL)	86.81 ± 4.9	83.93 ± 8.69	80–100
MCH (pg)	28.71 ± 1.95	27.16 ± 3.72	27–34
MCHC (g/dL)	33.06 ± 1.08	32.26 ± 1.67	30–36
RDW (%)	14.34 ± 3.8	15.58 ± 2.27	11.5–14.5
Creatinine (mg/dL)	0.67 ± 0.08	2.24 ± 1.62	0.7–1.2
GFR (mL/min/1.73 m^2^)	115.56 ± 16.97	38.5 ± 16.15	>60
HbA1c (%)	5.39 ± 0.22	7.49 ± 1.72	4–6
ACR (mg/g)		624.21 ± 170.85	<30
PCR (mg/g)		1428.92 ± 732.63	<150

## Data Availability

The data are available upon request from the authors.

## Supplementary Materials

The following supporting information can be downloaded at https://www.mdpi.com/article/10.3390/life15081312/s1: Table S1: Protein abbreviations and full names corresponding to proteins detected in diabetic nephropathy study; Table S2: Enrichment analysis of proteins in diabetic nephropathy patients compared to healthy controls.

## Abbreviations

The following abbreviations are used in this manuscript:

DN	Diabetic Nephropathy
DM	Diabetes Mellitus
DM1	Type 1 Diabetes
DM2	Type 2 Diabetes
DKD	Diabetic Kidney Disease
ESRD	End-Stage Renal Disease
GFR	Glomerular Filtration Rate
eGFR	Estimated Glomerular Filtration Rate
RAAS	Renin–Angiotensin–Aldosterone System
AGEs	Advanced Glycation End Products
Ang-II	Angiotensin II
ROS	Reactive Oxygen Species
IL-6	Interleukin-6
TNF-α	Tumor Necrosis Factor-Alpha
NF-κB	Nuclear Factor Kappa-Light-Chain-Enhancer of Activated B Cells
PKC	Protein Kinase C
MS	Mass Spectrometry
EDTA	Ethylenediaminetetraacetic Acid
PBS	Phosphate-Buffered Saline
FASP	Filter Aided Sample Preparation
LC-MS/MS	Liquid Chromatography–Mass Spectrometry/Mass Spectrometry
CE	Collision Energy
IMS	Ion Mobility Separation
KEGG	Kyoto Encyclopedia of Genes and Genomes
GO	Gene Ontology
DAVID	Database for Annotation, Visualization, and Integrated Discovery
SPSS	Statistical Package for the Social Sciences
PPI	Protein–Protein Interaction
STRING	Search Tool for the Retrieval of Interacting Genes/Proteins
WBC	White Blood Cell
RBC	Red Blood Cell
HGB	Hemoglobin
HCT	Hematocrit
PLT	Platelets
MCV	Mean Corpuscular Volume
MCH	Mean Corpuscular Hemoglobin
MCHC	Mean Corpuscular Hemoglobin Concentration
RDW	Red Cell Distribution Width
HbA1c	Hemoglobin A1c
ACR	Albumin-to-Creatinine Ratio
PCR	Protein-to-Creatinine Ratio
DEPs	Differentially Expressed Proteins
BP	Biological Processes
CC	Cellular Components
MF	Molecular Functions
HDL	High-Density Lipoprotein
HIF-2α	Hypoxia-Inducible Factor 2 Alpha
FSGS	Focal Segmental Glomerulosclerosis
CKD	Chronic Kidney Disease
vWF	von Willebrand Factor
LIS	Laboratory Information Systems
ELISA	Enzyme-linked Immunosorbent Assay
