# Supplementary material for: Exploring the Proteomic Signature of Diabetic Nephropathy: Implications for Early Diagnosis and Treatment
Source: Life (Basel). 2025 Aug 19;15(8):1312. doi: 10.3390/life15081312 (PMC12387283; doi:10.3390/life15081312)
Supplement: Supplementary file 1 [file life-15-01312-s001.zip › Supplementary Material 1 .pdf]

Supplementary Table 1 Protein Abbreviations and Full Names Corresponding to Proteins Detected in Diabetic Nephropathy Study. This table provides the full names of protein abbreviations used in the study, which are detected in diabetic nephropathy patients through proteomic analysis. The abbreviations correspond to protein identifiers found in the Human Protein Atlas database, accessible at https://www.proteinatlas.org.

Abbreviation	Full Name
A1BG	Alpha-1-B glycoprotein
A2M	Alpha-2-macroglobulin
ABCB5	ATP-binding cassette sub-family B member 5
ABCB9	ATP binding cassette subfamily B member 9
ACOXL	Acyl-CoA oxidase like
ACTB	Actin beta
ACTBL2	Actin beta like 2
ACTC1	Actin alpha cardiac muscle 1
ACTN1	Actinin alpha 1
ACTN4	Actinin alpha 4
ADAMTS13	ADAM metallopeptidase with thrombospondin type 1 motif 13
ADIPOQ	Adiponectin, C1Q and collagen domain containing
AFM	Afamin
AGAP11	Arf-GAP with GTPase, ANK repeat and PH domain-containing protein 11
AGT	Angiotensinogen
AHSG	Alpha-2-HS-glycoprotein
AIFM2	Apoptosis inducing factor mitochondria associated 2
ALB	Albumin
ALDOA	Aldolase, fructose-bisphosphate A
AMBP	Alpha-1-microglobulin/bikunin precursor
ANPEP	Alanyl aminopeptidase, membrane
ANKFY1	Ankyrin repeat and FYVE domain containing 1
ANKHD1	Ankyrin repeat and KH domain containing 1
ANKLE2	Ankyrin repeat and LEM domain containing 2
ANKRD20A1	Ankyrin repeat domain 20 family member A1
ANKRD30A	Ankyrin repeat domain 30A
ANKRD36C	Ankyrin repeat domain 36C
ANTXRL	Anthrax toxin receptor-like
APBB2	Amyloid beta precursor protein binding family B member 2
APCDD1	APC down-regulated 1
APCS	Amyloid P component, serum
APOA1	Apolipoprotein A1
APOA2	Apolipoprotein A2
APOA4	Apolipoprotein A4
APOB	Apolipoprotein B
APOC1	Apolipoprotein C1
APOC3	Apolipoprotein C3
APOC4	Apolipoprotein C4
APOC4-APOC2	APOC4-APOC2 readthrough

APOD	Apolipoprotein D
APOE	Apolipoprotein E
APOF	Apolipoprotein F
APOH	Apolipoprotein H
APOL1	Apolipoprotein L1
APOM	Apolipoprotein M
ARHGAP26	Rho GTPase activating protein 26
ARMCX2	Armadillo repeat containing X-linked 2
ATAD3A	ATPase family AAA domain containing 3A
ATRIP	ATR interacting protein
ATRN	Attractin
AZGP1	Alpha-2-glycoprotein 1, zinc-binding
B2M	Beta-2-microglobulin
BASP1	Brain abundant membrane attached signal protein 1
BCHE	Butyrylcholinesterase
BLMH	Bleomycin hydrolase
BLTP1	Bridge-like lipid transfer protein family member 1
BNC2	Basonuclin 2
BRCA1	BRCA1 DNA repair associated
BRCA2	BRCA2 DNA repair associated
BRPF1	Bromodomain and PHD finger containing 1
BRSK1	BR serine/threonine kinase 1
BTD	Biotinidase
C16orf78	Chromosome 16 open reading frame 78
C1QA	Complement C1q subcomponent subunit A
C1QB	Complement C1q subcomponent subunit B
C1QC	Complement C1q subcomponent subunit C
C1QTNF6	Complement C1q tumor necrosis factor-related protein 6
C1R	Complement C1r
C1RL	Complement C1r subcomponent like
C1S	Complement C1s
C2	Complement C2
C3	Complement C3
C4A	Complement C4A
C4BPA	Complement component 4 binding protein alpha
C4BPB	Complement component 4 binding protein beta
C5	Complement C5
C6	Complement C6
C7	Complement C7
C8A	Complement C8 alpha chain
C8B	Complement C8 beta chain
C8G	Complement C8 gamma chain
C9	Complement C9
CA1	Carbonic anhydrase 1

CA2	Carbonic anhydrase 2
CABP5	Calcium binding protein 5
CACNA1A	Calcium voltage-gated channel subunit alpha1 A
CALR	Calreticulin
CAMKK2	Calcium/calmodulin dependent protein kinase kinase 2
CAP1	Cyclase associated actin cytoskeleton regulatory protein 1
CARMIL3	Capping protein regulator and myosin 1 linker 3
CASP8	Caspase 8
CAT	Catalase
CC2D2A	Coiled-coil and C2 domain containing 2A
CCDC51	Coiled-coil domain containing 51
CCDC88A	Coiled-coil domain containing 88A
CCHCR1	Coiled-coil alpha-helical rod protein 1
CD14	CD14 molecule
CD163	CD163 molecule
CD44	CD44 molecule
CD5L	CD5 molecule like
CDH11	Cadherin 11
CDH5	Cadherin 5
CDK5RAP1	CDK5 regulatory subunit associated protein 1
CDK5RAP2	CDK5 regulatory subunit associated protein 2
CEP164	Centrosomal protein 164
CEP290	Centrosomal protein 290
CEP85L	Centrosomal protein 85 like
CES5A	Carboxylesterase 5A
CETP	Cholesteryl ester transfer protein
CFB	Complement factor B
CFD	Complement factor D
CFH	Complement factor H
CFHR1	Complement factor H related 1
CFHR2	Complement factor H related 2
CFI	Complement factor I
CHAF1A	Chromatin assembly factor 1 subunit A
CHD7	Chromodomain helicase DNA binding protein 7
CHL1	Cell adhesion molecule L1 like
CHTF18	Chromosome transmission fidelity factor 18
CIT	Citron rho-interacting serine/threonine kinase
CLEC3B	C-type lectin domain family 3 member B
CLIC1	Chloride intracellular channel 1
CLNK	Cytokine dependent hematopoietic cell linker
CLTC	Clathrin heavy chain
CLTCL1	Clathrin heavy chain like 1
CLU	Clusterin
CNDP1	Carnosine dipeptidase 1

COBLL1	Cordon-bleu WH2 repeat protein like 1
COG5	Component of oligomeric golgi complex 5
COL11A2	Collagen type XI alpha 2 chain
COL18A1	Collagen type XVIII alpha 1 chain
COMP	Cartilage oligomeric matrix protein
COQ8B	Coenzyme Q8B
CP	Ceruloplasmin
CPB2	Carboxypeptidase B2
CPLANE1	Ciliogenesis and planar polarity effector complex subunit 1
CPN1	Carboxypeptidase N subunit 1
CPN2	Carboxypeptidase N subunit 2
CROCC	Ciliary rootlet coiled-coil, rootletin
CRP	C-reactive protein
CRTAC1	Cartilage acidic protein 1
CST3	Cystatin-C
CTHRC1	Collagen triple helix repeat-containing protein 1
DAB2IP	Disabled homolog 2-interacting protein
DBH	Dopamine beta-hydroxylase
DCLK3	Doublecortin like kinase 3
DCUN1D5	Defective in cullin neddylation 1 domain containing 5
DDX42	Dead-box helicase 42
DDX5	Dead-box helicase 5
DENND3	DENN domain containing 3
DEPDC5	DEP domain containing 5, GATOR1 subcomplex subunit
DOCK10	Dedicator of cytokinesis 10
DOCK9	Dedicator of cytokinesis 9
DSG2	Desmoglein 2
ECM1	Extracellular matrix protein 1
EFEMP1	EGF containing fibulin extracellular matrix protein 1
EIF4A1	Eukaryotic translation initiation factor 4A1
ELF2	ETS-related transcription factor Elf-2
EML5	Echinoderm microtubule-associated protein-like 5
EPAS1	Endothelial PAS domain protein 1
EPB41L2	Erythrocyte membrane protein band 4.1 like 2
EPB41L3	Erythrocyte membrane protein band 4.1 like 3
EPHA10	Ephrin type-A receptor 10
F10	Coagulation factor X
F11	Coagulation factor XI
F12	Coagulation factor XII
F13A1	Coagulation factor XIII A chain
F13B	Coagulation factor XIII B chain
F2	Coagulation factor II
F5	Coagulation factor V
F7	Coagulation factor VII

F9	Coagulation factor IX
FAM13A	Family with sequence similarity 13 member A
FAM166B	Family with sequence similarity 166 member B
FAM177A1	Family with sequence similarity 177 member A1
FANCM	FA complementation group M
FARP1	FERM, ARH/RhoGEF and pleckstrin domain protein 1
FBLN1	Fibulin-1
FBXO22	F-box protein 22
FCGR3B	Fc gamma receptor IIIb
FCN2	Ficolin 2
FCN3	Ficolin 3
FERMT3	FERM domain containing kindlin 3
FETUB	Fetuin B
FGA	Fibrinogen alpha chain
FGB	Fibrinogen beta chain
FGD1	FYVE, RhoGEF and PH domain containing 1
FGD4	FYVE, RhoGEF and PH domain containing 4
FGG	Fibrinogen gamma chain
FGL1	Fibrinogen like 1
FILIP1	Filamin A interacting protein 1
FLNA	Filamin A
FMN2	Formin 2
FN1	Fibronectiın 1
FNDC3A	Fibronectin type III domain containing 3A
FSD1	Fibronectin type III and SPRY domain containing 1
FUBP1	Far upstream element binding protein 1
FXR1	FMR1 autosomal homolog 1
G6PD	Glucose-6-phosphate dehydrogenase
GAPDH	Glyceraldehyde-3-phosphate dehydrogenase
GAS7	Growth arrest specific 7
GBF1	Golgi brefeldin A resistant guanine nucleotide exchange factor 1
GC	GC vitamin D binding protein
GCC2	GRIP and coiled-coil domain containing 2
GGH	Gamma-glutamyl hydrolase
GIMAP1-GIMAP5	GIMAP1-GIMAP5 readthrough
GIPC2	GIPC PDZ domain containing family member 2
GLYATL1	Glycine-N-acyltransferase like 1
GNPDA1	Glucosamine-6-phosphate deaminase 1
GOLGA2	Golgin A2
GOLGA3	Golgin A3
GOLGB1	Golgin B1
GP1BA	Glycoprotein Ib platelet subunit alpha
GPLD1	Glycosylphosphatidylinositol specific phospholipase D1
GPX3	Glutathione peroxidase 3

GRID1	Glutamate ionotropic receptor delta type subunit 1
GSN	Gelsolin
GUK1	Guanylate kinase 1
HABP2	Hyaluronan binding protein 2
HARS1	Histidyl-tRNA synthetase 1
HBA2	Hemoglobin subunit alpha 2
HBB	Hemoglobin subunit beta
HERC3	HECT and RLD domain containing E3 ubiquitin protein ligase 3
HERC4	HECT and RLD domain containing E3 ubiquitin protein ligase 4
HGFAC	Hepatocyte growth factor activator
HLA-A	HLA class I histocompatibility antigen, A alpha chain
HP	Haptoglobin
HPR	Haptoglobin-related protein
HPX	Hemopexin
HRG	Histidine rich glycoprotein
HS6ST3	Heparan sulfate 6-O-sulfotransferase 3
HSPA1A	Heat shock protein family A (Hsp70) member 1A
HTT	Huntingtin
HYDIN	Hydrocephalus-inducing protein homolog
ICAM3	Intercellular adhesion molecule 3
ICAM5	Intercellular adhesion molecule 5
IDH3G	Isocitrate dehydrogenase [NAD] subunit gamma, mitochondrial
IFIT5	Interferon-induced protein with tetratricopeptide repeats 5
IGFALS	Insulin-like growth factor-binding protein complex acid labile subunit
IGFBP3	Insulin-like growth factor-binding protein 3
IGHA1	Immunoglobulin heavy constant alpha 1
IGHA2	Immunoglobulin heavy constant alpha 2
IGHG1	Immunoglobulin heavy constant gamma 1 (G1m marker)
IGHG2	Immunoglobulin heavy constant gamma 2 (G2m marker)
IGHG4	Immunoglobulin heavy constant gamma 4 (G4m marker)
IGHM	Immunoglobulin heavy constant mu
IGHV3OR16-12	Immunoglobulin heavy variable 3/OR16-12 (non-functional)
IGHV3OR16-9	Immunoglobulin heavy variable 3/OR16-9 (non-functional)
IGKC	Immunoglobulin kappa constant
IGKV1-8	Immunoglobulin kappa variable 1-8
IGKV2D-24	Immunoglobulin kappa variable 2D-24 (non-functional)
IGKV3-20	Immunoglobulin kappa variable 3-20
IGKV4-1	Immunoglobulin kappa variable 4-1
IGLC6	Immunoglobulin lambda constant 6
IQCJ-SCHIP1	IQCJ-SCHIP1 readthrough
IQGAP2	IQ motif containing GTPase activating protein 2
IREB2	Iron responsive element binding protein 2
ITIH1	Inter-alpha-trypsin inhibitor heavy chain H1
ITIH2	Inter-alpha-trypsin inhibitor heavy chain H2

ITIH3	Inter-alpha-trypsin inhibitor heavy chain H3
ITIH4	Inter-alpha-trypsin inhibitor heavy chain H4
JCHAIN	Joining chain of multimeric IgA and IgM
KCNIP4	Potassium voltage-gated channel interacting protein 4
KCNMA1	Potassium calcium-activated channel subfamily M alpha 1
KIF11	Kinesin family member 11
KIF13B	Kinesin family member 13B
KIN	Kin17 DNA and RNA binding protein
KITLG	KIT ligand
KLHL3	Kelch like family member 3
KLKB1	Kallikrein B1
KNG1	Kininogen 1
L1CAM	L1 cell adhesion molecule
LAD1	Ladinin 1
LARP1	La ribonucleoprotein 1, translational regulator
LBP	Lipopolysaccharide binding protein
LCAT	Lecithin-cholesterol acyltransferase
LCORL	Ligand dependent nuclear receptor corepressor like
LCP1	Lymphocyte cytosolic protein 1
LDHA	Lactate dehydrogenase A
LDHB	Lactate dehydrogenase B
LGALS3BP	Galectin 3 binding protein
LIMA1	LIM domain and actin binding 1
LIN52	Lin-52 DREAM MuvB core complex component
LPA	Lipoprotein(a)
LRG1	Leucine rich alpha-2-glycoprotein 1
LRPPRC	Leucine rich pentatricopeptide repeat containing
LRRC45	Leucine rich repeat containing 45
LRRC9	Leucine rich repeat containing 9
LRRK2	Leucine rich repeat kinase 2
LUC7L2	LUC7 like 2, pre-mRNA splicing factor
LUM	Lumican
LYZ	Lysozyme
MAD1L1	Mitotic arrest deficient 1 like 1
MAP2	Microtubule associated protein 2
MAP2K3	Mitogen-activated protein kinase kinase 3
MAP3K19	Mitogen-activated protein kinase kinase kinase 19
MAP3K2	Mitogen-activated protein kinase kinase kinase 2
MAP4	Microtubule associated protein 4
MAP9	Microtubule associated protein 9
MASP1	MBL associated serine protease 1
MASP2	MBL associated serine protease 2
MBL2	Mannose binding lectin 2
MCAM	Melanoma cell adhesion molecule

MCL1	MCL1 apoptosis regulator, BCL2 family member
MED14	Mediator of RNA polymerase II transcription subunit 14
MFSD2A	Major facilitator superfamily domain containing 2A
MICA	MHC class I polypeptide-related sequence A
MIS18BP1	MIS18 binding protein 1
MN1	MN1 proto-oncogene, transcriptional regulator
MOK	MOK protein kinase
MPDZ	Multiple PDZ domain crumbs cell polarity complex component
MPP1	MAGUK p55 scaffold protein 1
MRPS27	Mitochondrial ribosomal protein S27
MST1	Macrophage stimulating 1
MYH10	Myosin heavy chain 10
MYH14	Myosin heavy chain 14
MYO6	Myosin VI
MYPN	Myopalladin
MYZAP	Myocardial zonula adherens protein
NARS2	Asparaginyl-tRNA synthetase 2, mitochondrial
NBEAL1	Neurobeachin like 1
NCAPH	Non-SMC condensin I complex subunit H
NCOR1	Nuclear receptor corepressor 1
NECAP2	NECAP endocytosis associated 2
NEDD1	NEDD1 gamma-tubulin ring complex targeting factor
NLN	Neurolysin
NLRP5	NLR family pyrin domain containing 5
NNT	Nicotinamide nucleotide transhydrogenase
NOL3	Nucleolar protein 3
NSD1	Nuclear receptor binding SET domain protein 1
NUMA1	Nuclear mitotic apparatus protein 1
NUP58	Nucleoporin p58/p45
NXPE3	NXPE family member 3
OFD1	OFD1 centriole and centriolar satellite protein
OLFML2B	Olfactomedin like 2B
OPLAH	5-oxoprolinase, ATP-hydrolysing
ORM1	Orosomucoid 1
PAAF1	Proteasomal ATPase associated factor 1
PAG1	Phosphoprotein membrane anchor with glycosphingolipid microdomains 1
PARP10	Poly(ADP-ribose) polymerase family member 10
PBXIP1	PBX homeobox interacting protein 1
PCDH1	Protocadherin 1
PCDHA11	Protocadherin alpha 11
PCNT	Pericentrin
PCYOX1	Prenylcysteine oxidase 1
PDE4DIP	Phosphodiesterase 4D interacting protein
PEPD	Peptidase D

PFN1	Profilin 1
PGLYRP2	Peptidoglycan recognition protein 2
PHLDB1	Pleckstrin homology like domain family B member 1
PI16	Peptidase inhibitor 16
PIEZO2	Piezo type mechanosensitive ion channel component 2
PIKFYVE	Phosphoinositide kinase, FYVE-type zinc finger containing
PIP4K2C	Phosphatidylinositol-5-phosphate 4-kinase type 2 gamma
PKM	Pyruvate kinase M1/2
PKN2	Protein kinase N2
PLCG1	Phospholipase C gamma 1
PLG	Plasminogen
PLTP	Phospholipid transfer protein
PLXNB2	Plexin-B2
PMFBP1	Polyamine-modulated factor 1-binding protein 1
PON1	Serum paraoxonase/arylesterase 1
PON3	Serum paraoxonase/lactonase 3
POTEF	POTE ankyrin domain family member F
POTEI	POTE ankyrin domain family member I
POTEKP	Putative beta-actin-like protein 3
PPBP	Pro-platelet basic protein
PPFIA2	PTPRF interacting protein alpha 2
PPFIBP1	PPFIA binding protein 1
PPM1A	Protein phosphatase, Mg2+/Mn2+ dependent 1A
PPT1	Palmitoyl-protein thioesterase 1
PRDX2	Peroxiredoxin 2
PRG4	Proteoglycan 4
PROC	Protein C, inactivator of coagulation factors Va and VIIIa
PROS1	Protein S
PROZ	Protein Z, vitamin K dependent plasma glycoprotein
PRPS2	Phosphoribosyl pyrophosphate synthetase 2
PRR11	Proline rich 11
PTGDS	Prostaglandin D2 synthase
PTPRF	Protein tyrosine phosphatase receptor type F
PZP	PZP alpha-2-macroglobulin like
QSOX1	Quiescin sulfhydryl oxidase 1
RBP4	Retinol binding protein 4
RCN1	Reticulocalbin 1
RERE	Arginine-glutamic acid dipeptide repeats
RPGRIP1L	RPGRIP1 like
RPL32	Ribosomal protein L32
RPRD1B	Regulation of nuclear pre-mRNA domain containing 1B
RPS6KC1	Ribosomal protein S6 kinase C1
S100A8	S100 calcium binding protein A8
S100A9	S100 calcium binding protein A9

SAA1	Serum amyloid A1
SAA2	Serum amyloid A2
SAA2-SAA4	SAA2-SAA4 readthrough
SAMSN1	SAM domain, SH3 domain and nuclear localization signals 1
SAPCD1	Suppressor APC domain containing 1
SBF1	SET binding factor 1
SBNO2	Strawberry notch homolog 2
SCIN	Scinderin
SELENOP	Selenoprotein P
SELL	Selectin L
SEMA4G	Semaphorin 4G
SERPINA1	Serpin family A member 1
SERPINA10	Serpin family A member 10
SERPINA3	Serpin family A member 3
SERPINA4	Serpin family A member 4
SERPINA5	Serpin family A member 5
SERPINA6	Serpin family A member 6
SERPINA7	Serpin family A member 7
SERPINC1	Serpin family C member 1
SERPIND1	Serpin family D member 1
SERPINF1	Serpin family F member 1
SERPINF2	Serpin family F member 2
SERPING1	Serpin family G member 1
SGCZ	Sarcoglycan zeta
SH3GLB1	SH3 domain containing GRB2 like, endophilin B1
SHANK2	SH3 and multiple ankyrin repeat domains 2
SHANK3	SH3 and multiple ankyrin repeat domains 3
SHBG	Sex hormone binding globulin
SLC26A6	Solute carrier family 26 member 6
SMARCAD1	SWI/SNF-related, matrix-associated actin-dependent regulator of chromatin, subfamily a, containing DEAD/H box 1
SMC2	Structural maintenance of chromosomes 2
SNX19	Sorting nexin 19
SORBS2	Sorbin and SH3 domain containing 2
SPAG5	Sperm associated antigen 5
SPATS2	Spermatogenesis associated serine rich 2
SPEF2	Sperm flagellar 2
SPEN	Spen family transcriptional repressor
SPP2	Secreted phosphoprotein 2
SPTB	Spectrin beta, erythrocytic
SRRM4	Serine/arginine repetitive matrix 4
SSBP3	Single stranded DNA binding protein 3
STAG3	Stromal antigen 3
STX19	Syntaxin 19

SUN3	Sad1 and UNC84 domain containing 3
SYTL2	Synaptotagmin like 2
TAGLN2	Transgelin 2
TATDN1	TatD DNase domain containing 1
TBC1D2	TBC1 domain family member 2
TBC1D4	TBC1 domain family member 4
TBCD	Tubulin folding cofactor D
TBCEL	Tubulin folding cofactor E like
TCEAL1	Transcription elongation factor A like 1
TCF20	Transcription factor 20
TCF4	Transcription factor 4
TEX13A	Testis expressed 13A
TF	Trasnferrin
TFRC	Transferrin receptor
TGDS	TDP-glucose 4,6-dehydratase
TGFBI	Transforming growth factor beta induced
THBS1	Thrombospondin 1
TIAM1	TIAM Rac1 associated GEF 1
TLN1	Talin 1
TLN2	Talin 2
TMCC1	Transmembrane and coiled-coil domain family 1
TNRC6C	Trinucleotide repeat containing adaptor 6C
TPM3	Tropomyosin 3
TPM4	Tropomyosin 4
TRAF4	TNF receptor associated factor 4
TRAV19	T cell receptor alpha variable 19
TRIM15	Tripartite motif containing 15
TRIOBP	TRIO and F-actin binding protein
TRMT11	TRNA methyltransferase 11 homolog
TRPM3	Transient receptor potential cation channel subfamily M member 3
TTC21B	Tetratricopeptide repeat domain 21B
TTLL11	Tubulin tyrosine ligase like 11
TTR	Transthyretin
TUBA1B	Tubulin alpha 1b
TULP1	TUB like protein 1
UBE3A	Ubiquitin protein ligase E3A
USP10	Ubiquitin Specific Peptidase 10
USP40	Ubiquitin Specific Peptidase 40
USP49	Ubiquitin Specific Peptidase 49
VASN	Vasorin
VCAM1	Vascular Cell Adhesion Molecule 1
VCL	Vinculin
VNN1	Vanin 1
VTN	Vitronectin

VWF	Von Willebrand factor
WBP1L	WW domain binding protein 1 like
WDR27	WD repeat domain 27
WHAMM	WASP homolog associated with actin, golgi membranes and microtubules
XPC	XPC complex subunit, DNA damage recognition and repair factor
XRCC3	X-ray repair cross complementing 3
YARS1	Tyrosyl-tRNA synthetase 1
YWHAZ	Tyrosine 3-Monooxygenase/Tryptophan 5-Monooxygenase Activation Protein Z
ZC3H12A	Zinc finger CCCH-type containing 12A
ZDHHC17	Zinc finger DHHC-type palmitoyltransferase 17
ZNF131	Zinc Finger Protein 131
ZNF417	Zinc Finger Protein 417
ZNF484	Zinc Finger Protein 484
ZNF638	Zinc Finger Protein 638
ZNF830	Zinc Finger Protein 830
ZSWIM5	Zinc finger SWIM-type containing 5
ZYX	Zyxin
