# Supplementary material for: Exploring the Proteomic Signature of Diabetic Nephropathy: Implications for Early Diagnosis and Treatment
Source: Life (Basel). 2025 Aug 19;15(8):1312. doi: 10.3390/life15081312 (PMC12387283; doi:10.3390/life15081312)
Supplement: Supplementary file 1 [file life-15-01312-s001.zip › Supplementary Material 2.pdf]

Supplementary Table 2 Enrichment Analysis of Proteins in Diabetic Nephropathy Patients Compared to Healthy Controls. This table presents the results of the enrichment analysis for proteins identified in the DN group compared to healthy controls. The table includes gene counts, the genes involved in each pathway, fold enrichment values, and the corresponding p-values, highlighting key pathways associated with DN-related molecular alterations.

**Term**	**Gene count**	**Genes in each pathway from our result**	**Fold Enrichment**	**P-value**
Complement and coagulation cascades	5	FGA, C6, FGG, A2M, SERPINA5	12	6.90E-04
Platelet activation	5	FGA, ADAMTS13, FGG, TLN2, TLN1	8.4	2.60E-03
Regulation of actin cytoskeleton	5	PIKFYVE, C6, ACTN1, PIP4K2C, IQGAP2	4.6	2.10E-02
Cytoskeleton in muscle cells	5	ZYX, DSG2, TLN2, TLN1, MYPN	4.5	2.20E-02
Shigellosis	5	ACTN1, PLCG1, TLN2, TLN1, CD44	4.2	2.80E-02
Cell adhesion molecules	4	SELL, ICAM3, L1CAM, PTPRF	5.3	3.60E-02
Inositol phosphate metabolism	3	PIKFYVE, PLCG1, PIP4K2C	8.7	4.50E-02
Platelet degranulation	8	FGA, AHSG, ACTN1, ALB, FGG, APOA1, A2M, TLN1	10.6	8.40E-06
Response to elevated platelet cytosolic Ca2+	8	FGA, AHSG, ACTN1, ALB, FGG, APOA1, A2M, TLN1	10.2	1.10E-05
Hemostasis	14	FGA, AHSG, ACTN1, FGG, APOA1, L1CAM, SERPINA5, ADAMTS13, SELL, ALB, PLCG1, A2M, TLN1, CD44	3.9	4.20E-05
Post-translational protein phosphorylation	6	FGA, AHSG, ALB, FGG, APOA1, APOE	9.5	3.80E-04
Regulation of Insulin-like Growth Factor (IGF) transport and uptake by Insulin-like Growth Factor Binding Proteins (IGFBPs)	6	FGA, AHSG, ALB, FGG, APOA1, APOE	8.2	7.30E-04
Platelet activation. signaling and aggregation	8	FGA, AHSG, ACTN1, ALB, FGG, APOA1, A2M, TLN1	5.2	7.40E-04
Innate Immune System	16	FGA, AHSG, FGG, ICAM3, IQGAP2, LYZ, CPN1, FCGR3B, CASP8, LRG1, C6, SELL, PLCG1, B2M, CEP290, CD44	2.6	7.60E-04
HDL remodeling	3	ALB, APOA1, APOE	51.5	1.40E-03
Formation of Fibrin Clot (Clotting Cascade)	4	FGA, FGG, A2M, SERPINA5	17.6	1.40E-03
Retinoid metabolism and transport	4	RBP4, APOM, APOA1, APOE	15.6	2.00E-03
Recruitment of NuMA to mitotic centrosomes	5	NUMA1, CEP164, PCNT, CEP290, CDK5RAP2	9.1	2.00E-03
Anchoring of the basal body to the plasma membrane	5	CEP164, RPGRIP1L, PCNT, CEP290, CDK5RAP2	8.8	2.40E-03

Metabolism of fat-soluble vitamins	4	RBP4, APOM, APOA1, APOE	14.3	2.60E-03
GRB2:SOS provides linkage to MAPK signaling for Integrins	3	FGA, FGG, TLN1	34.3	3.20E-03
p130Cas linkage to MAPK signaling for integrins	3	FGA, FGG, TLN1	34.3	3.20E-03
Amyloid fiber formation	5	FGA, APOA1, APOE, LYZ, B2M	7.8	3.60E-03
Plasma lipoprotein assembly	3	APOA1, APOE, A2M	27.1	5.20E-03
Metabolism of vitamins and cofactors	6	PARP10, RBP4, APOM, BTD, APOA1, APOE	5.1	5.70E-03
Neutrophil degranulation	9	FCGR3B, LRG1, SELL, AHSG, IQGAP2, LYZ, B2M, CEP290, CD44	3.2	5.80E-03
Mitotic Prometaphase	6	NUMA1, CEP164, PCNT, CEP290, SMC2, CDK5RAP2	5	6.30E-03
Common Pathway of Fibrin Clot Formation	3	FGA, FGG, SERPINA5	23.4	7.00E-03
Extracellular matrix organization	7	FGA, EFEMP1, ACTN1, FGG, ICAM3, A2M, CD44	4	7.30E-03
Loss of Nlp from mitotic centrosomes	4	CEP164, PCNT, CEP290, CDK5RAP2	9.8	7.50E-03
Loss of proteins required for interphase microtubule organization from the centrosome	4	CEP164, PCNT, CEP290, CDK5RAP2	9.8	7.50E-03
AURKA Activation by TPX2	4	CEP164, PCNT, CEP290, CDK5RAP2	9.4	8.40E-03
Plasma lipoprotein assembly. remodeling. and clearance	4	ALB, APOA1, APOE, A2M	9.2	9.10E-03
Integrin signaling	3	FGA, FGG, TLN1	19.1	1.00E-02
Recruitment of mitotic centrosome proteins and complexes	4	CEP164, PCNT, CEP290, CDK5RAP2	8.4	1.20E-02
Centrosome maturation	4	CEP164, PCNT, CEP290, CDK5RAP2	8.4	1.20E-02
Integrin cell surface interactions	4	FGA, FGG, ICAM3, CD44	8.1	1.30E-02
Regulation of PLK1 Activity at G2/M Transition	4	CEP164, PCNT, CEP290, CDK5RAP2	7.8	1.40E-02
Plasma lipoprotein remodeling	3	ALB, APOA1, APOE	15.1	1.60E-02
Signaling by high-kinase activity BRAF mutants	3	FGA, FGG, TLN1	14.3	1.80E-02
Platelet Aggregation (Plug Formation)	3	FGA, FGG, TLN1	13.2	2.10E-02
Visual phototransduction	4	RBP4, APOM, APOA1, APOE	6.7	2.10E-02
MAP2K and MAPK activation	3	FGA, FGG, TLN1	12.9	2.20E-02
Binding and Uptake of Ligands by Scavenger Receptors	3	ALB, APOA1, APOE	12.3	2.40E-02
Signaling by RAF1 mutants	3	FGA, FGG, TLN1	12	2.50E-02

Cilium Assembly	5	CEP164, RPGRIP1L, PCNT, CEP290, CDK5RAP2	4.3	2.80E-02
Paradoxical activation of RAF signaling by kinase inactive BRAF	3	FGA, FGG, TLN1	11	3.00E-02
Signaling downstream of RAS mutants	3	FGA, FGG, TLN1	11	3.00E-02
Signaling by moderate kinase activity BRAF mutants	3	FGA, FGG, TLN1	11	3.00E-02
Signaling by RAS mutants	3	FGA, FGG, TLN1	11	3.00E-02
Immune System	19	FGA, FANCM, AHSG, FGG, IFIT5, ICAM3, KLHL3, IQGAP2, LYZ, CPN1, FCGR3B, CASP8, LRG1, C6, SELL, PLCG1, B2M, CEP290, CD44	1.6	3.60E-02
Post-translational protein modification	15	FGA, USP49, DCUN1D5, COG5, AHSG, EPAS1, FGG, APOA1, KLHL3, XPC, KIN, FCGR3B, ADAMTS13, ALB, APOE	1.7	3.90E-02
Metabolism of proteins	19	FGA, HARS1, USP49, DCUN1D5, COG5, AHSG, EPAS1, FGG, TBCD, APOA1, KLHL3, XPC, LYZ, KIN, FCGR3B, ADAMTS13, ALB, APOE, B2M	1.6	4.40E-02
Cell surface interactions at the vascular wall	4	SELL, PLCG1, L1CAM, CD44	5	4.40E-02
HDL assembly	2	APOA1, A2M	42.9	4.50E-02
